# Supplementary material for: A 2D hybrid perovskite ferroelectric with switchable polarization and photoelectric robustness down to monolayer
Source: Nat Commun. 2025 Mar 28;16:3028. doi: 10.1038/s41467-025-58164-z (PMC11953369; doi:10.1038/s41467-025-58164-z)
Supplement: Supplementary file 1 — Supplementary Information [file 41467_2025_58164_MOESM1_ESM.pdf]

Supplementary Information for

A van der Waals hybrid perovskite ferroelectric semiconductor with switchable polarization and superior photoelectric robustness down to monolayer

Yuzhong Hu[1,2*], Haidong Lu[3], Shehr Bano Masood[3], Clemens Göhler[2], Shangpu Liu[4], Alexei Gruverman[3] and Marin Alexe[1,*]

[1]Department of Physics, The University of Warwick, Coventry CV4 7AL, UK.

[2]Institute for Molecular Systems Engineering and Advanced Materials, Heidelberg University, 69120, Heidelberg, Germany

[3]Department of Physics and Astronomy, University of Nebraska Lincoln, NE 68588, USA

[4]Physikalisch-Chemisches Institut, Universität Heidelberg, Im Neuenheimer Feld 229, 69120 Heidelberg, Germany

*Corresponding author. Email: huyu0012@e.ntu.edu.sg (Y.H.); m.alexe@warwick.ac.uk (M.A.

This PDF file includes:

Supplementary Notes 1 - 2

Supplementary Figures 1 - 10

Supplementary References

Supplement Note 1 | Extraction of energy band gap by photoconductivity spectra

The band gap of semiconductor materials can be estimated from photocurrent spectra by the Tauc plot method which is widely applied in various 2D systems[1-3]. Assuming the measured photocurrent (I_p) is proportional to the absorption coefficient α near band edge, for 2D systems, the band gap E_g can be extracted by the following equation[3-5]:

$$(I_p \times hv)^b = C(hv - E_g) \quad (1)$$

where hv is the photon energy and C is a constant. The exponent b equals to 2 or 1/2 for direct and indirect optical transition, respectively. The band gap can be determined from the x-axis intercept of the plot of $(I_p \times hv)^b$ vs. hv.

Using this method, we extracted the band gap of a bulk crystal sample at different temperatures (Fig. 3a) as well as flake samples with different thicknesses (Fig. 4c). Here, we present the bulk and monolayer cases to show E_g extraction and to make comparison. Supplementary Fig. 1a shows the photocurrent spectrum of these two samples at room temperature. Fig. S1b presents the Tauc plot of bulk crystals with b = 2 and 1/2 to estimate its effective band gap by direct and indirect optical transitions. The extracted values of 1.93 and 1.86 eV show good agreement with the reported band gap of 1.8 eV[6], while the small difference between these two indicates a relatively small amount of indirect optical transition. By the same method, the direct and indirect band gap of 2.33 and 2.21 eV were obtained for the monolayer sample (Supplementary Fig. 1c). The larger difference reflects an enhancement in indirect transition, which is consistent with the more obvious tail feature near the absorption edge of the monolayer photocurrent spectrum (see Supplementary Fig. 1a).

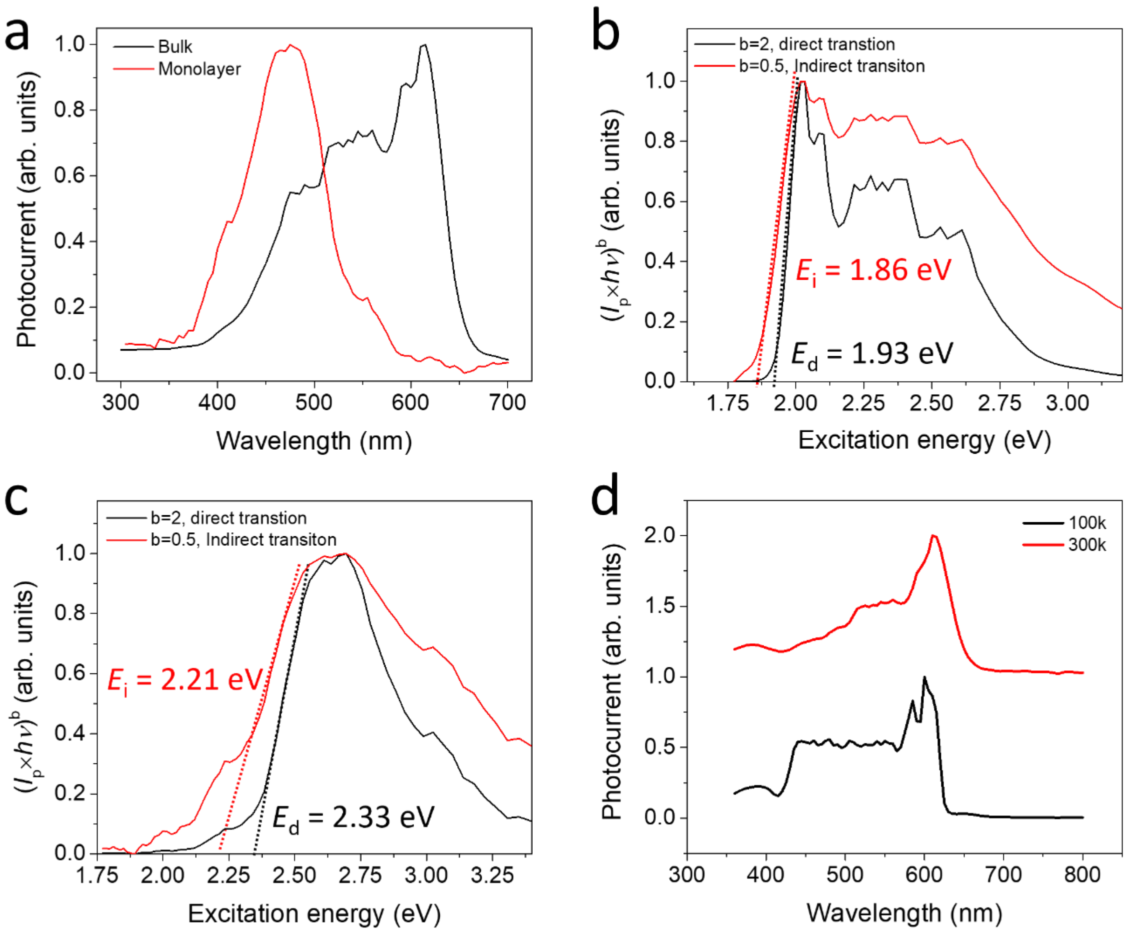

Supplementary Figure 1 | Photoconductivity spectrum and related analysis. a, Photoconductivity spectrum of a bulk crystal and monolayer flake of PEPI under monochromatic illumination with an electric bias of 2V. **b** and **c**, Plot of $(I_{pc} \cdot h\nu)^b$ vs. $h\nu$ to extract band gap values for bulk (**b**) and monolayer (**c**) samples. **d**, Photoconductivity spectra at 100K and 300K. As discussed in the main text, the increased photoresponse in short-wavelength region of the 100 K spectrum suggests a decrease in surface recombination.

Supplementary Note 2 | Analysis of Hetch equation for 2D flake system

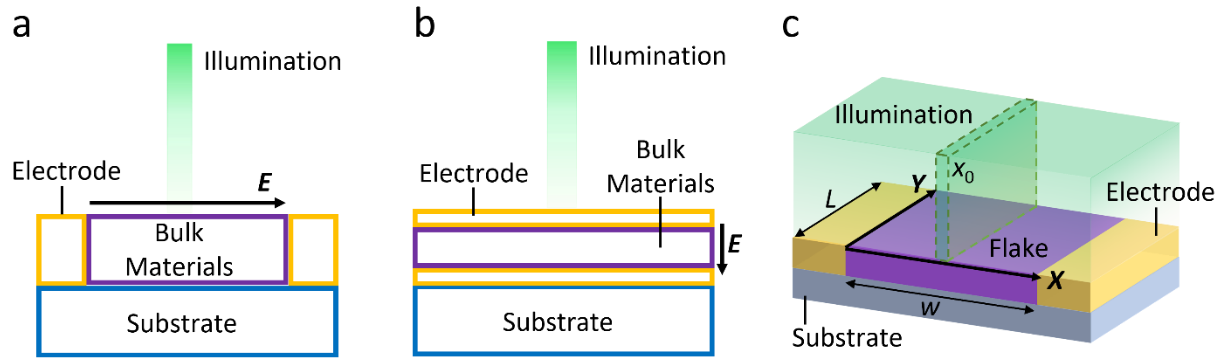

Supplementary Figure 2 | Different photoelectric systems for $\mu\tau$ measurements. Structures of bulk devices with illumination direction normal **(a)** and parallel **(b)** to the electric field (**E**). **c**, Physics parameters, coordinates and illumination of flake device.

Here we analyze and discuss the Hetch equation for nanodevice in detail. In general, there are two Hetch equations widely used for $\mu\tau$ estimation regarding different photoelectric systems. The first one is employed in systems where the illumination direction is normal to the electric field (Supplementary Fig. 2a)[7,8]:

$$I = \frac{I_0 \mu\tau V}{w^2}\left[1 - \exp(\frac{-w^2}{\mu\tau V})\right] \tag{2}$$

Another case is when the electric field is parallel to the illumination direction, and the illumination is generally non-uniform across the material thickness direction because of the finite penetration depth of light. In such device, with a structure shown in Supplementary Fig. 2b, the $\mu\tau$ is evaluated using the following equation[9,10]:

$$I = \frac{I_0 \mu \tau V}{T^2} \cdot \frac{1 - \exp(-\frac{T^2}{\mu \tau V})}{1 + \frac{Tv}{V\mu}} \quad (3)$$

Here I, I_0, μ, τ, V, w, T and v are photocurrent, saturated photocurrent, carrier mobility, carrier lifetime, applied voltage, distance between electrodes, sample thickness and surface recombination velocity, respectively.

A 2D flake with in-plane electrodes has a device structure similar to the one sketched in Fig. S2a described by the equation 2. However, with uniform illumination across the device, it does not fulfill the boundary condition that carriers are generated by an illumination point/strip with a much smaller width compared to that of the device area[11,12]. The size of a 2D flake device is commonly around 10 μm, which is much smaller than a generic non-focused laser spots that can have diameters of several hundred microns. With the 2D device positioned at the center of the laser beam, the illumination is quasi-uniform with photocarriers uniformly generated throughout the device area. In this specific case, the drifting length of the photocarriers that reach the electrode is position-dependent, which is quite different from the original assumption (that the drifting length is identical for all carriers and equals the distance between the electrode and the illumination point/strip) applied in the deduction of the classical Hetch equation. Under this condition, a modified Hetch equation is required for $\mu\tau$ estimation. Here we discuss and develop such a modified Hetch equation that can be employed to roughly estimate the $\mu\tau$ in nanoflake systems from photoresponse-voltage characteristics.

For ease of calculation, the device is assumed to be cuboid in shape and uniformly illuminated, as shown in Supplementary Fig. 2c. Generally, the absorption coefficient for PbI-based hybrid perovskites in the visible region is around 10^4-10^5cm^{-1} [13-15]. This yields a penetration depth of few hundreds nanometers[16]. Given the small thickness of the PEPI flakes, it is reasonable to assume

uniform light absorption and consequently an uniform photo-generated carrier density across the sample thickness direction.

We further assume N to be the number of photons reaching the device during a short time and the quantum efficiency of the material to be η. For a narrow illumination strip with a width of dx_0 at position x_0, the photo-induced carrier dQ_0 at local position x_0 is given by:

$$dQ_0 = Ne \cdot \frac{A_1}{A_2} \cdot \eta = Ne \cdot \frac{Ldx_0}{wL} \cdot \eta = \frac{\eta Ne}{w} dx_0 \quad (4)$$

Where w, L, e, A_1 and A_2 are the width of flake (distance between the electrodes), length of the electrode, elementary charge, area of the strip and area of the device, respectively. According to Lenard relationship[12], under electric bias V, the photo-induced carriers by light strip will distribute across the device with their number decreasing exponentially along the drifting distance due to effects such as recombination and limited carrier lifetime. The number of carriers at position x can be expressed as:

$$dQ(x) = dQ_0 \cdot \exp\left(-\frac{(x_0 - x)w}{\mu\tau V}\right) = \frac{\eta Ne}{w} dx_0 \exp\left(-\frac{(x_0 - x)w}{\mu\tau V}\right) \quad (5)$$

For an inter-electrodes distance of w, the effective charging at the electrode due to elementary charge motion over a small distance S can be calculated by[12]:

$$q = e \cdot \frac{S}{w} \quad (6)$$

The effective charging at the electrode position induced by this illumination strip can thus be obtained by integrating along the drifting direction:

$$dQ_{x0} = \int dQ_x \cdot \frac{dx}{w}$$

$$= \frac{\mu\tau V\eta Ne}{w^3} dx_0(1 - \exp(\frac{x_0 w}{\mu\tau V}))$$

Assuming the incident number of photons within the illuminated strip per unit of time is $dN/dt = \Phi$, the current density can be calculated as:

$$dJ(x_0) = \frac{1}{TL} dQ_{x0}/dt$$

$$= \frac{\mu\tau V\eta\Phi e}{w^3 TL} dx_0(1 - \exp(\frac{x_0 w}{\mu\tau V})) \quad (7)$$

where T is the sample thickness. This gives the photocurrent generated by the light strip at a certain position x_0, which is comparable to the classic Hetch equation (eq. 2). The total photocurrent J produced by the uniform illumination in the flake device can thus be obtained by integrating the strip across the x direction:

$$J = \int dJ(x_0)$$

$$= \int_0^w \frac{\mu\tau V\eta\Phi e}{w^3 TL} \left(1 - \exp\left(\frac{x_0 w}{\mu\tau V}\right)\right) dx_0$$

$$= \frac{\mu\tau V\eta\Phi e}{w^2 TL} \left(1 + \frac{\mu\tau V}{w} \cdot \exp\left(-\frac{w^2}{\mu\tau V}\right) - \frac{\mu\tau V}{w^2}\right) \quad (8)$$

With specific device parameters, the $\mu\tau$ product can be roughly estimated by fitting the J-V characteristics with the above equation.

Supplementary Figures

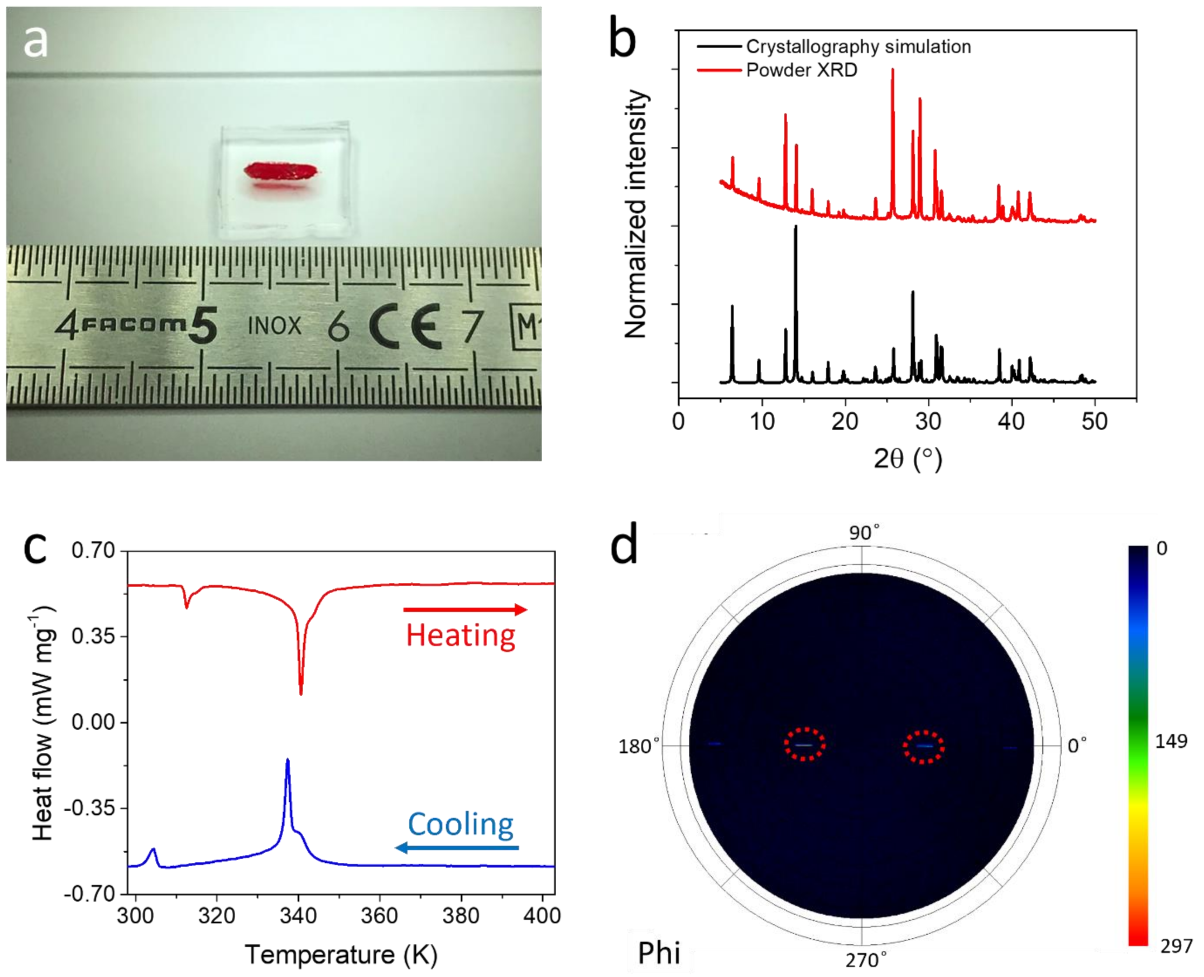

Supplementary Figure 3 | Photo, powder XRD, DSC characterization and pole figure of PEPI. **a**, Photo of a typical PEPI bulk single crystal. **b**, Experimental and crystallographic simulated XRD patterns. **c**, DSC characterization along heating and cooling cycles. **d**, A typical pole figure of a PEPI bulk crystal. The peaks around Phi = 0 and 180 degree (in red dotted circles) indicate the position of the [17 1 1] peaks that help to identify the [0 1 1] crystal orientation in real space. The sharp and strong peaks indicate the high quality of the crystal. The four polarization directions (along [0 0 1] and [0 1 0]) can thus be identified at Phi = 45, 135, 225 and 315 degrees.

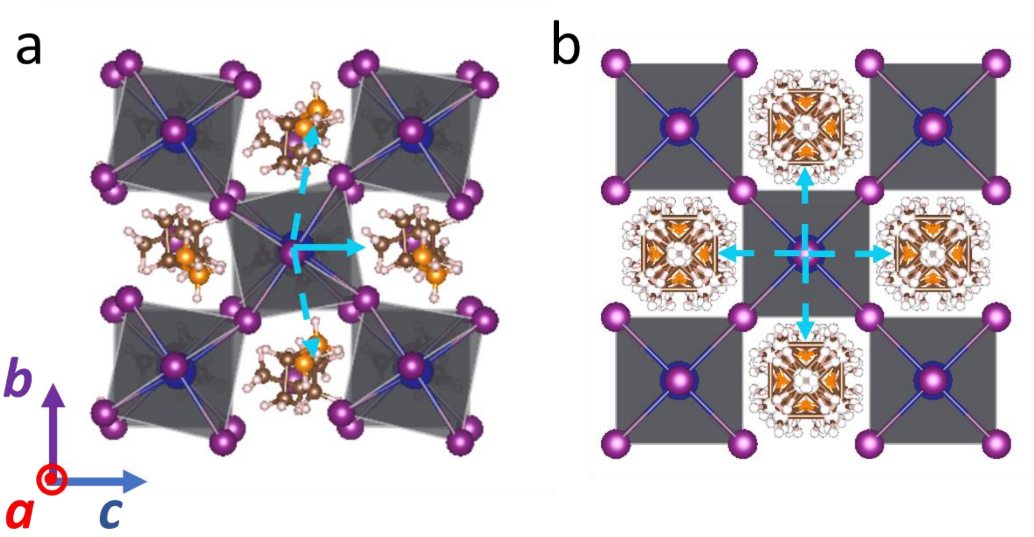

Supplementary Figure 4 | Crystallographic structure and dipole at ferroelectric (a) and paraelectric (b) phases. Crystallographic data are from a previous report[6]. Crystal structures and spontaneous polarization viewed along the *a*-axis in the (**a**) ferroelectric and (**b**) paraelectric phase. PEPI is a biaxial ferroelectric material with polarization along the *b* and *c* axes. Similar to other organic-inorganic hybrid ferroelectrics, a simple polarization analysis can be made by regarding N^+ and the center of the PbI_6^{2-} octahedron (Pb sites) as the centers of cations and anions. The paraelectric phase of PEPI possesses mirror symmetry along the *b* and *c* axes with fourfold orientationally disordered organic cations. After transferring to ferroelectric phase, the cations are frozen in one of four equivalent off-center positions, resulting in net in-plane polarization along the symmetry-breaking directions (*b* and *c* axes). Fig. 4a shows a projection along the *c* direction, which can be switched by an external electric field.

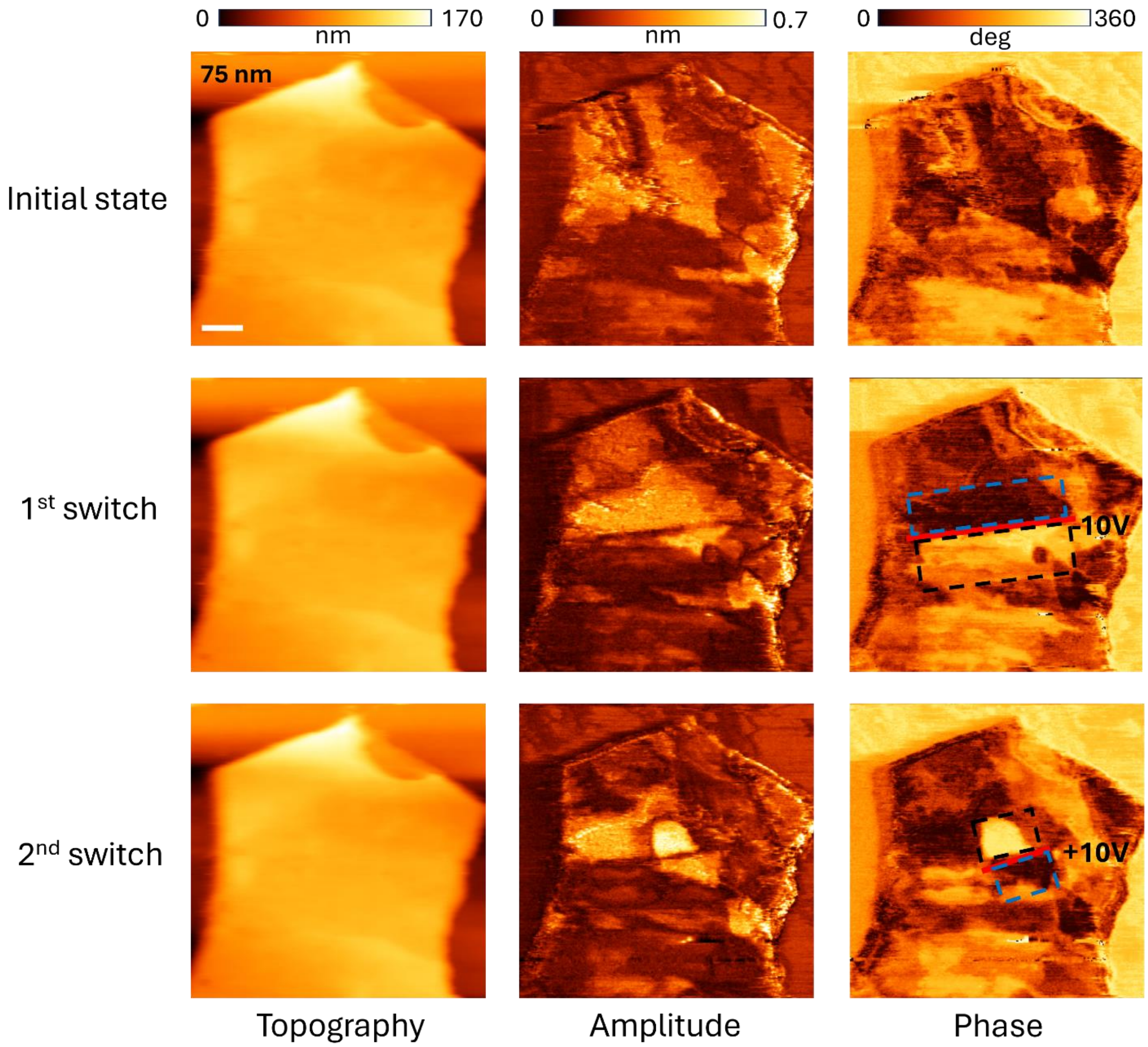

Supplementary Figure 5 | PFM images of polarization switching of a 75 nm PEPI flake. Diagrams were obtained using the Asylum system in resonant mode. The scale bar is 1 μm. The color scales in each type of signal (topography, amplitude and phase) are unified. The same applies to Figure S6-8.

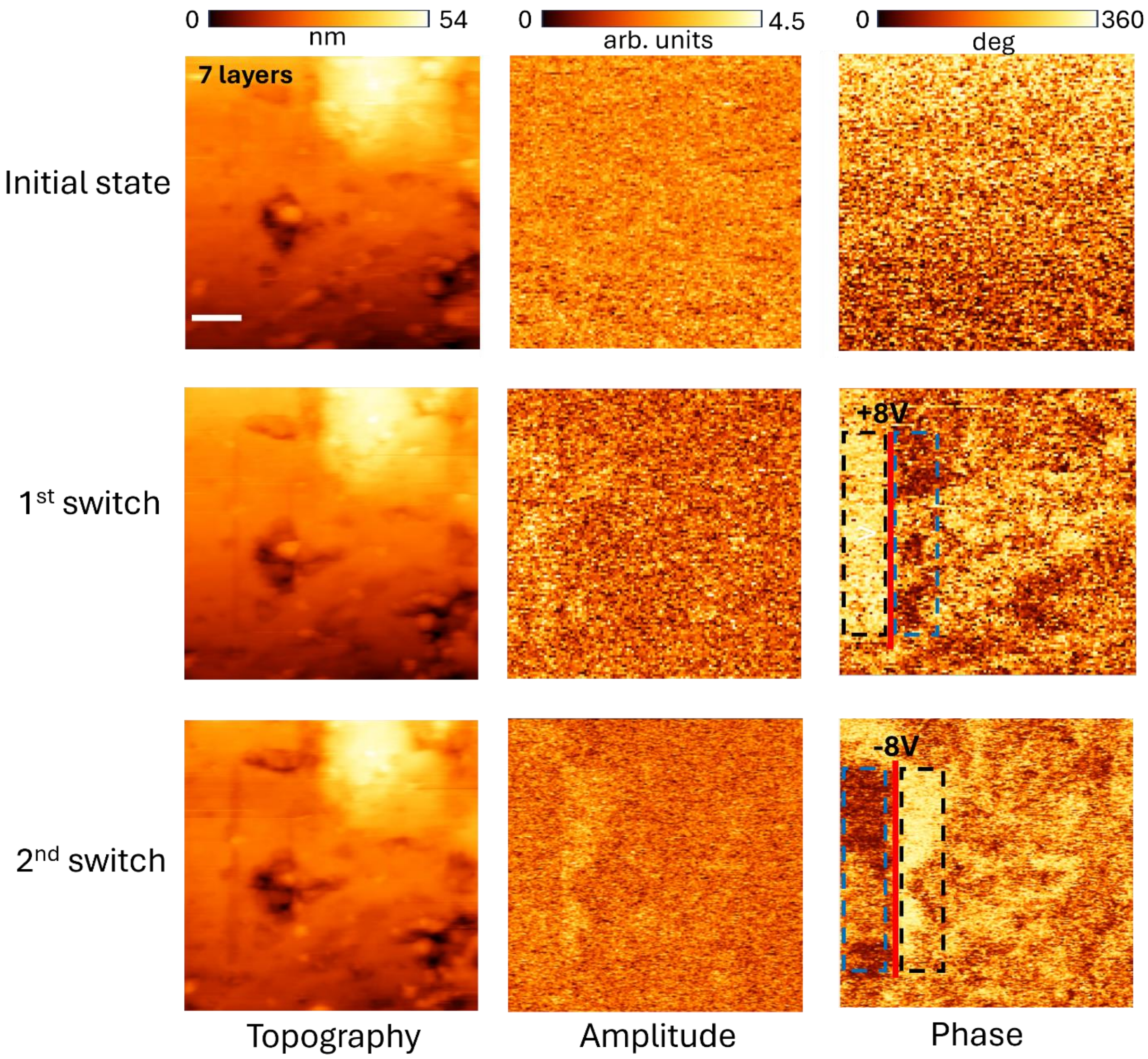

Supplementary Figure 6 | PFM images of polarization switching of a 7-layer PEPI flake. Diagrams were obtained using the XE-100 Park in non-resonant mode. The scale bar is 1 µm.

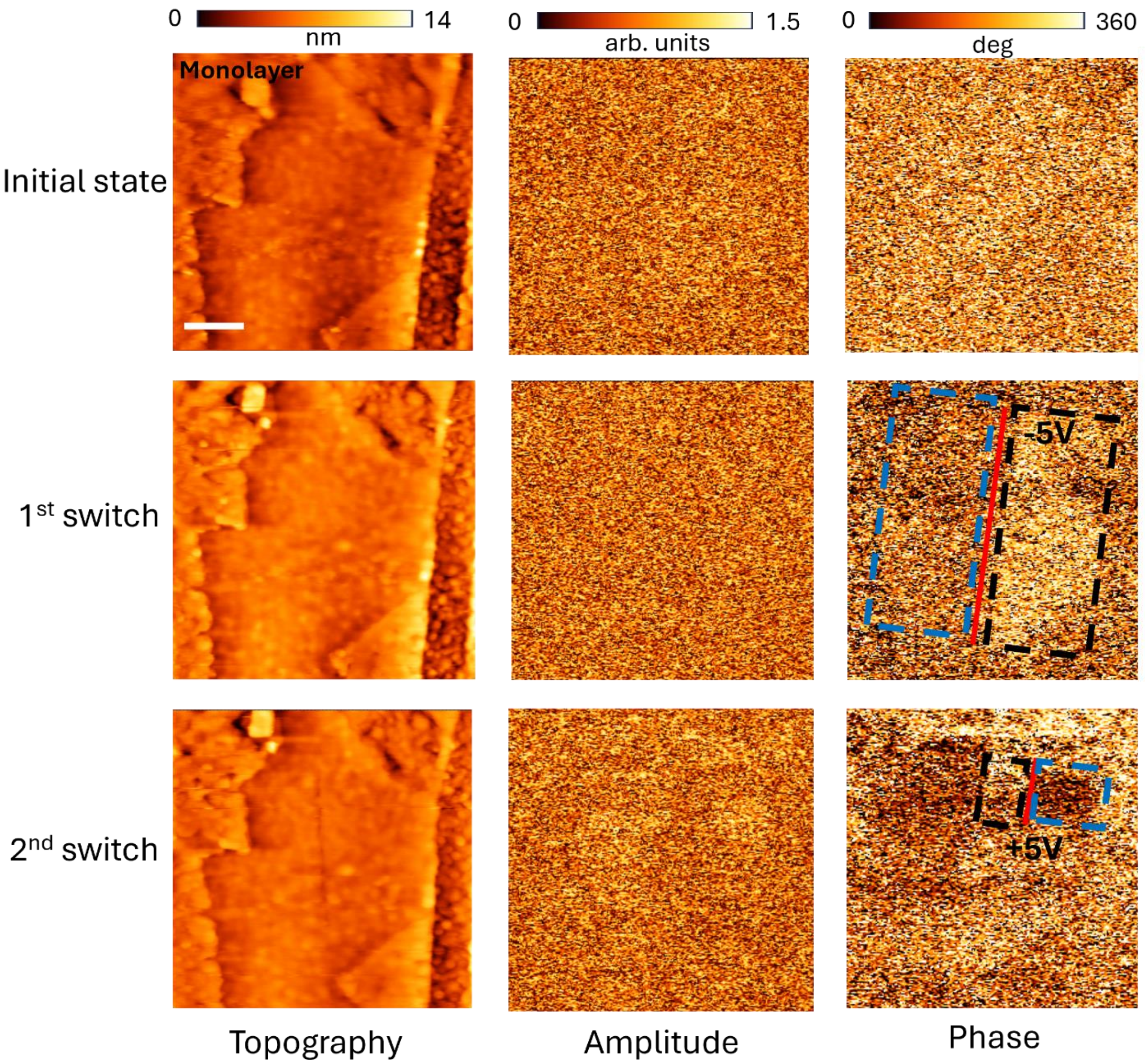

Supplementary Figure 7 | PFM images of polarization switching of a monolayer PEPI flake.

Measured by the XE-100 Park. The scale bar is 0.5 μm.

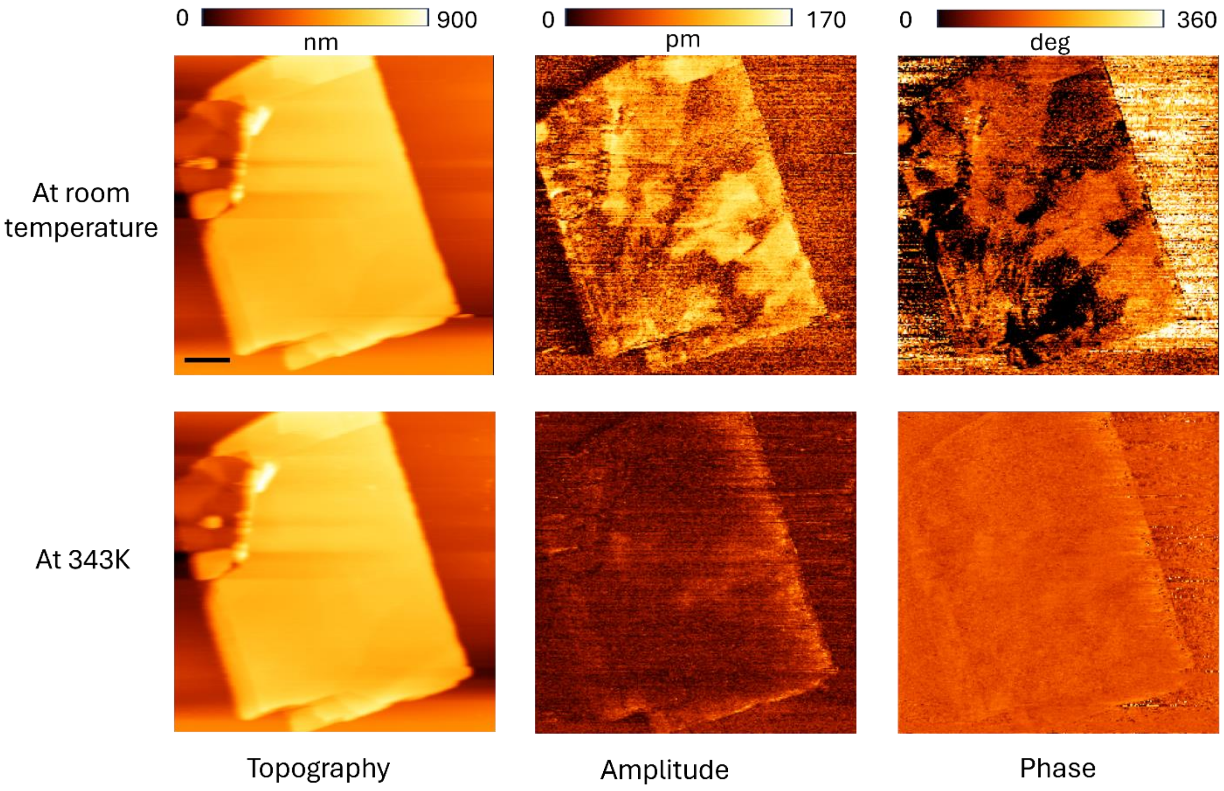

Supplementary Figure 8 | Temperature-dependent PFM study of a 400 nm PEPI film. PEPI is in the ferroelectric phase at room temperature and transitions to the paraelectric phase at 343 K. Measured by the Asylum system. The scale bar is 4 μm.

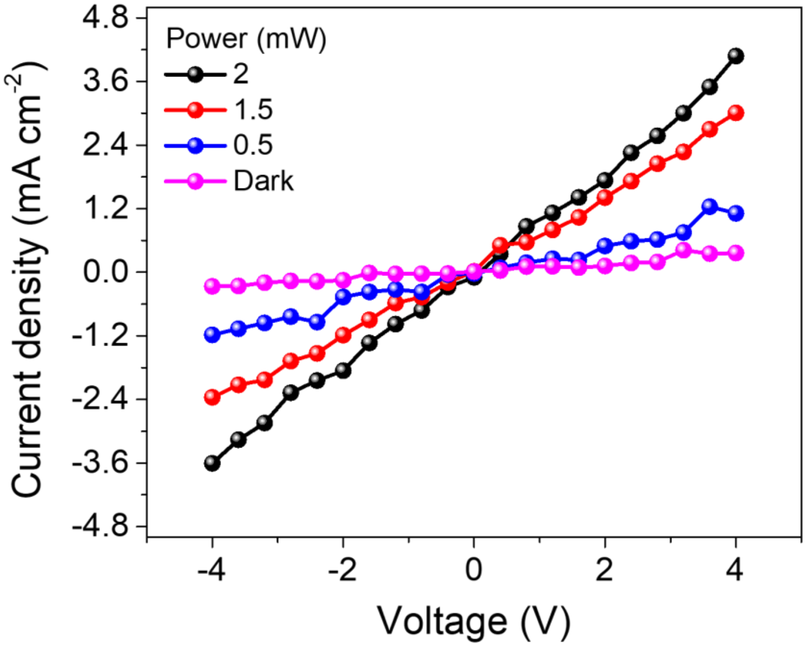

Supplementary Figure 9 | *J-V* curves of a monolayer PEPI under illumination with 520 nm laser at different power.

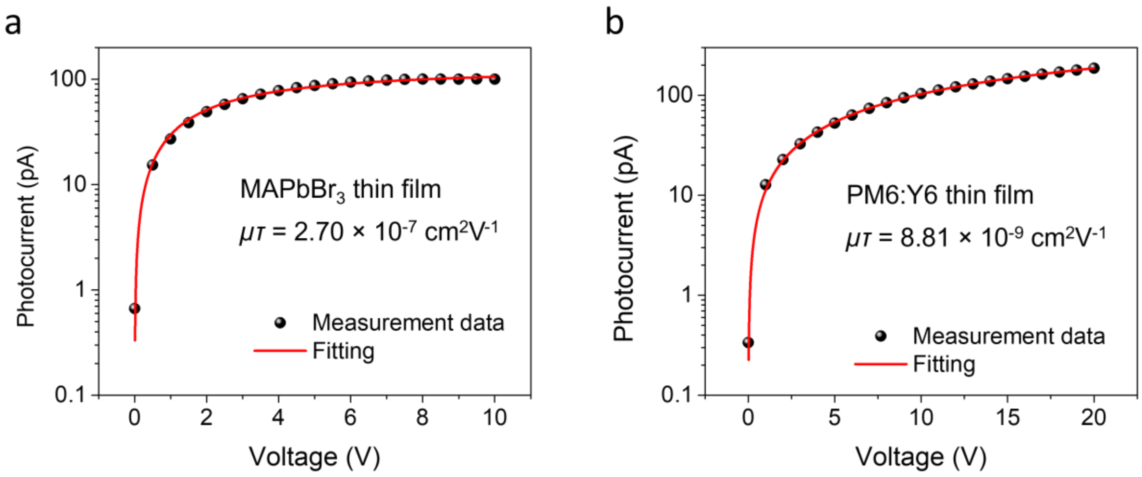

Supplementary Figure 10 | Photocurrent-voltage characteristics of spin-coated (a) $MAPbBr_3$ and (b) PM6:Y6 thin films. The employed in-plane structure is the same as that of the PEPI nanoflakes.

Supplementary References

1 Quereda, J., Ghiasi, T. S., van Zwol, F. A., van der Wal, C. H. & van Wees, B. J. Observation of bright and dark exciton transitions in monolayer MoSe2 by photocurrent spectroscopy. *2D Materials* **5**, 015004, doi:10.1088/2053-1583/aa8aa0 (2018).

2 Lei, S. *et al.* Evolution of the Electronic Band Structure and Efficient Photo-Detection in Atomic Layers of InSe. *ACS Nano* **8**, 1263-1272, doi:10.1021/nn405036u (2014).

3 Shlyakhov, I. *et al.* Measurement of direct and indirect bandgaps in synthetic ultrathin MoS2 and WS2 films from photoconductivity spectra. *J. Appl. Phys.* **129**, doi:10.1063/5.0046305 (2021).

4 Kam, K. K. & Parkinson, B. A. Detailed photocurrent spectroscopy of the semiconducting group VIB transition metal dichalcogenides. *The Journal of Physical Chemistry* **86**, 463-467, doi:10.1021/j100393a010 (1982).

5 Patil, P. D., Wasala, M., Ghosh, S., Lei, S. & Talapatra, S. Broadband photocurrent spectroscopy and temperature dependence of band gap of few-layer indium selenide (InSe). *Emergent Materials* **4**, 1029-1036, doi:10.1007/s42247-021-00248-9 (2021).

6 Han, S. *et al.* Tailoring of a visible-light-absorbing biaxial ferroelectric towards broadband self-driven photodetection. *Nat. Commun.* **12**, 284, doi:10.1038/s41467-020-20530-4 (2021).

7 Ji, C. *et al.* Monolayer-to-Multilayer Dimensionality Reconstruction in a Hybrid Perovskite for Exploring the Bulk Photovoltaic Effect Enables Passive X-ray Detection. *Angew. Chem. Int. Ed.* **60**, 20970-20976, doi:https://doi.org/10.1002/anie.202108145 (2021).

8 Yakunin, S. *et al.* Detection of gamma photons using solution-grown single crystals of hybrid lead halide perovskites. *Nature Photonics* **10**, 585-589, doi:10.1038/nphoton.2016.139 (2016).

9 Wei, H. *et al.* Sensitive X-ray detectors made of methylammonium lead tribromide perovskite single crystals. *Nature Photonics* **10**, 333-339, doi:10.1038/nphoton.2016.41 (2016).

10 Many, A. High-field effects in photoconducting cadmium sulphide. *J. Phys. Chem. Solids* **26**, 575-578, doi:https://doi.org/10.1016/0022-3697(65)90133-2 (1965).

11 Auden, E. C., Vizkelethy, G., Serkland, D. K., Bossert, D. J. & Doyle, B. L. Modeling charge collection efficiency degradation in partially depleted GaAs photodiodes using the 1- and 2-carrier Hecht equations. *Nuclear Instruments and Methods in Physics Research Section B: Beam Interactions with Materials and Atoms* **399**, 12-19, doi:https://doi.org/10.1016/j.nimb.2017.03.045 (2017).

12 Hecht, K. Zum Mechanismus des lichtelektrischen Primärstromes in isolierenden Kristallen. *Z. Phys.* **77**, 235-245, doi:10.1007/BF01338917 (1932).

13 Fujiwara, H., Kato, M., Tamakoshi, M., Miyadera, T. & Chikamatsu, M. Optical Characteristics and Operational Principles of Hybrid Perovskite Solar Cells. *physica status solidi (a)* **215**, 1700730, doi:https://doi.org/10.1002/pssa.201700730 (2018).

14 Zhang, Q., Chu, L., Zhou, F., Ji, W. & Eda, G. Excitonic Properties of Chemically Synthesized 2D Organic–Inorganic Hybrid Perovskite Nanosheets. *Adv. Mater.* **30**, 1704055, doi:https://doi.org/10.1002/adma.201704055 (2018).

15 Ma, S. *et al.* Two-dimensional organic-inorganic hybrid perovskite: from material properties to device applications. *Science China Materials* **61**, 1257-1277, doi:10.1007/s40843-018-9294-5 (2018).

16 De Wolf, S. *et al.* Organometallic Halide Perovskites: Sharp Optical Absorption Edge and Its Relation to Photovoltaic Performance. *J. Phys. Chem. Lett* **5**, 1035-1039, doi:10.1021/jz500279b (2014).
